# Lichenoid drug eruption associated with Bendamustine

**DOI:** 10.1038/bcj.2016.48

**Published:** 2016-06-24

**Authors:** Y Kusano, Y Terui, M Yokoyama, K Hatake

**Affiliations:** 1 Department of Hematology and Oncology, Cancer Institute Hospital, Japanese Foundation for Cancer Research, Tokyo, Japan

A 65-year-old man had relapsed follicular lymphoma. When he suffered a fifth relapse, he received a regimen containing bendamustine and rituximab (BR). Four weeks later, he presented with systemic erythema, swelling and bullous lesions of lips, the oral cavity, andnasal mucosa and eye pain. He could not open his mouth and had severe pain. Steven–Johnson syndrome/toxic epidermal necrolysis was diagnosed. With corticosteroid treatment and intravenous administration of oxycodone, he recovered for 2 weeks and was discharged. However, 10 days later, bullous lesions appeared again at lips (Figure 1a), oral cavity and genital mucosa. In addition, numerous erythematous plaques with Koebner's phenomenon were observed on his trunk (Figure 1b). Blood test, in which antibody was measured, showed neither candidiasis nor pemphigus. Microscopic examination revealed interface dermatitis with necrotic keratinocytes, which was a histological feature of lichenoid drug eruption. Prednisone 5 mg daily was initiated for his lichenoid drug eruption. Although with 4-month treatment his manifestation recovered little by little, mucosal lesions were intractable. His lymphoma was observed afterward because he achieved partial response by one cycle of BR. One year later, however, his wife became aware of his changing mental status. He eventually died of central nervous system involvement in lymphoma.

Lichenoid drug eruption, also known as drug-induced lichen planus, is an uncommon cutaneous adverse effect of several drugs.^1, 2^ It is characterized by a symmetric eruption resembling lichen planus on the trunk and extremities. Lichenoid drug eruption may sometimes be difficult to differentiate from Stevens–Johnson syndrome/toxic epidermal necrolysis, in particular, at the time of onset.^3^ However, interface dermatitis is found in histological appearance, resulting in difference between lichenoid drug eruption and Steven–Johnson syndrome.^4^ In general, lichenoid drug eruptions resolve spontaneously in a few weeks to a few months with the discontinuation of the offending drug.^5^ On the other hand, in our case, systemic corticosteroid was refractory to eruptions. Although the mechanism of lichenoid drug reaction has been unknown, lichenoid drug eruption is thought to be associated with activation of CD8 autocytotoxic T lymphocytes against epidermal cells. Bendamustine induced prolonged lymphocytopenia, inparticular CD4 T lymphocytes. There was no understanding between bendamustine and uncommon clinical course of lichenoid drug eruption in our case. To the best our knowledge, this is a first report of lichenoid drug eruption associated with bendamustine.

## Figures and Tables

**Figure 1:**
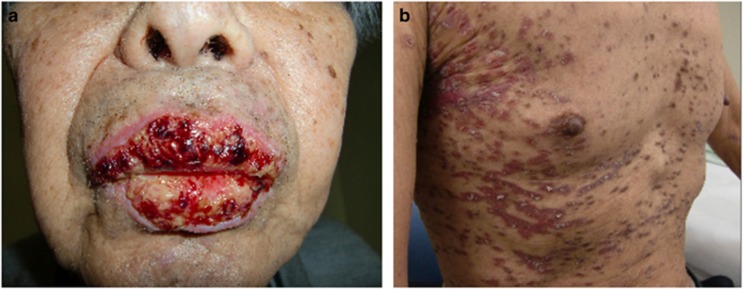
(**a**) Bullous lesions at lips. (**b**) Erythematous plaques with Koebner's phenomenon on his trunk.
